# Stage specific immune responses to schistosomes may explain conflicting results in malaria-schistosome coinfection studies

**DOI:** 10.1016/j.idm.2025.05.008

**Published:** 2025-05-20

**Authors:** Sarah Rollason, Eleanor Riley, Joanne Lello

**Affiliations:** a School of Biosciences, Cardiff University, Cardiff, UK; b University of Edinburgh, Edinburgh, UK

**Keywords:** Coinfection, *Schistosoma mansoni*, *Schistosoma haematobium*, *Plasmodium falciparum*, Agent-based modelling

## Abstract

Malaria and schistosomiasis are two of the most clinically important human parasitic diseases in terms of morbidity and mortality, collectively causing approximately 800,000 deaths annually. Coinfection with their causative parasites, *Plasmodium* spp. and *Schistosoma* spp., is common, particularly in sub-Saharan Africa. These parasites may interact with each other via their effects on the host immune system, but studies to date report conflicting consequences of such interactions, some suggesting that schistosomes are associated with reduced parasitaemia in malaria infection while others report increased parasitaemia. Schistosomes stimulate different immune components in early versus late infection. Using agent-based modelling we explore whether stage of infection could be a factor explaining the conflicting coinfection outcomes. Effects of schistosomes on blood stage malaria were modelled by adjusting the immune components within the model according to the response provoked by each schistosome stage. We find the dynamics of malaria infections are greatly influenced by the stage of schistosomes, with acute and chronic schistosome infections having opposite effects on both peak infected erythrocyte counts and duration. Our findings offer a possible explanation for the apparent contradictions between studies and highlight the importance of considering the stage of schistosome infection when exploring the relationship between these two parasites.

## 1 Introduction

Annually, around 190 million people worldwide are infected with *Schistosoma* spp. (Murray et al., 2020) and 241 million with malaria (World Health Organization, 2021). These infections are co-endemic in large areas of the tropics and coinfection with both parasites is common (Degarege et al., 2016; Donohue et al., 2019). The interactions between schistosomes and malaria have not been clearly elucidated, with different studies presenting conflicting outcomes both in terms of host health and parasite dynamics. In terms of human health, increases in usome studies (Anchang-Kimbi et al., 2017; Bustinduy et al., 2013; Degarege et al., 2012; Deribew et al., 2013; Friis et al., 2000; Getie et al., 2015; Kinung'hi et al., 2017; Mduluza-Jokonya et al., 2020; Okafor & Elenwo, 2007; Sumbele, Otia, Bopda, et al., 2021; Tuasha et al., 2019; S. Wilson, Vennervald, et al., 2007), while reduction in the incidence and severity of complications has been seen in others (Briand et al., 2005; Doumbo et al., 2014; Kinung'hi et al., 2014; Lyke et al., 2005; Sumbele, Otia, Francis, et al., 2021; Tokplonou et al., 2020). Similarly, in relation to parasite dynamics, increased malaria parasitaemia and prevalence have been reported (Doumbo et al., 2014; Florey et al., 2012; Friis et al., 2000; Midzi et al., 2008; Reilly et al., 2008; Samuels et al., 2012; Sangweme et al., 2010; Tuasha et al., 2019), but so has reduced parasitaemia (Briand et al., 2005; Kinung'hi et al., 2014; Lemaitre et al., 2014; Lyke, Dabo, et al., 2012; Lyke et al., 2005; McDowell et al., 2022; Tokplonou et al., 2020). The majority of studies exploring coinfection of malaria and schistosomes have looked at the effects in school age children or adults and cover a wide age range. Few studies have focused on coinfection in pre-school age children (Bejon et al., 2008; Green et al., 2011; McDowell et al., 2022; Mduluza-Jokonya et al., 2020).

Pre-school age children (PSAC) are known to be the age group most at risk from malaria, carrying the majority of the burden of mortality. Of over 600,000 deaths attributed to malaria annually, 73% occur in children aged under 5 years (World Health Organization, 2020). The burden of schistosomiasis in this age group is also increasingly recognised (Musuva et al., 2017; Sircar et al., 2018; Stothard et al., 2013; Stothard, Sousa-Figueiredo, Betson, Green, et al., 2011; Verani et al., 2011). The burden of severe and fatal malaria in PSAC makes the need to understand the mechanism and consequences of coinfection in this group critical, especially given the recent inclusion of PSAC in mass drug administration for schistosome control (World Health Organization, 2022).

Malaria and schistosomes can interact with each other via their effects on the host immune system. During infection, T cell differentiation drives the host immune response and is crucial in determining host-parasite dynamics. Microparasites, like malaria, largely promote a type 1 biased immune response. This immune response is characterised by inflammation, fever, and early clearance of the microparasite and the production of the typical cytokines IFN-γ and IL-12 by a host of immune cells including Th1, NK, γδ T and CD8^+^ T cells (Perez-Mazliah & Langhorne, 2015; Torre et al., 2002). The early innate immune response promoted by these cytokines is crucial in controlling peak parasitaemia via cell-mediated killing (Stevenson & Riley, 2004). Conversely, macroparasites, such as helminths, generally provoke a response strongly biased toward a type 2 response, characterised by the activation of wound healing pathways involving M2 macrophages and the production of cytokines such as IL-4, IL-5 and IL-13 from immune cell types including Th2 and Th2-T follicular helper (Th2-Tfh) cells (Gause et al., 2013; Henry et al., 2017).

Both Th1 and Th2-associated cytokines can drive B cell maturation and antibody production, although with differences in the predominant IgG subclass and isotypes produced (Garraud et al., 2003). Cytophilic IgG1 and IgG3 antibodies are the isotypes primarily associated with clinical protection against malaria in humans (Cavanagh et al., 2001; Diallo et al., 2010; Dodoo et al., 2008; Groux & Gysin, 1990; Lusingu et al., 2005; Soe et al., 2004; Tongren et al., 2006). The contributions of T cell lineages and their associated cytokines to this antibody-mediated protection from malaria is not completely understood and involves interplay between multiple arms of the immune system (Kurup et al., 2019). Th1 cytokines have been implicated in the production of protective antibodies but also in pathological downregulation of antibody-producing B cells (Hansen et al., 2017; Obeng-Adjei et al., 2015; Ryg-Cornejo et al., 2016; Sebina et al., 2016). With regards to Th2 cytokines, while it has been demonstrated that an antibody defence to malaria can be mounted in the absence of IL-4 (von der Weid et al., 1994), IL-4 production does play a role in upregulation of the humoral immune response to malaria (Sahoo et al., 2016). The major producer of IL-4 in helminth infection is Th2-Tfh (Chen et al., 2014; King & Mohrs, 2009; Zaretsky et al., 2009), which are indispensable in antibody-associated control of malaria (Pérez-Mazliah et al., 2017; Soon et al., 2021). Th2-Tfh proliferation and elevated IL-4 are correlated with higher IgG1 antibodies titres (Chan et al., 2020, 2022; King & Mohrs, 2009; Oyong et al., 2022; Troye-Blomberg et al., 1990), while inhibition results in reduced titres (Liu et al., 2003; Turqueti-Neves et al., 2014). Moreover, as these Th1 and Th2-associated cytokines promote differentiation down their own pathway while cross suppressing each other (Fernandez-Botran et al., 1986; Fiorentino et al., 1991; Gajewski & Fitch, 1988), the cytokine profile driven by one parasite can affect the immune response to a second parasite during coinfection (Mabbott, 2018). A third T helper profile is important in both microparasitic and macroparasitic infections. Regulatory T cells (Tregs) produce cytokines which downregulate both type 1 and type 2 immune responses (Belkaid & Rouse, 2005; Sakaguchi, 2003; Walker et al., 2003), thereby preventing excessive or prolonged T cell activation.

Schistosomes provoke different cytokine profiles depending on their life cycle stage with acute infection producing a mixed Th1/Th2 profile (Langenberg et al., 2020; Silveira-Lemos et al., 2013) and strongly polarising to a more classic helminth-driven Th2 profile later in infection, during egg production (Kaplan et al., 1998; M. Wilson, Vennervald, et al., 2007). Adult female worms produces hundreds of highly immunogenic eggs per day, and around half of these become trapped in host tissues, providing an ongoing source of antigen and becoming the focal point of granulomatous type 2 immune reactions (Costain et al., 2018). This antigenic input is sufficient to cause an overall bias of the immune system towards a type 2 response (M. Wilson, Vennervald, et al., 2007). Later in the course of chronic infection this is accompanied by regulatory cytokines, hypothesised to be protective against the fibrosis associated with long-term exposure to schistosome eggs (Baumgart et al., 2006; Taylor et al., 2006; Watanabe et al., 2007). Due to the changing overall profile of T helper cell polarisation over the course of schistosome infection, coinfecting malarial parasites may face different host immune profiles, depending on the stage of the schistosome life cycle at which the coinfection occurs. In turn these different immune profiles may influence malaria dynamics.

There is a relative scarcity of data on T cell populations and their associated cytokines during schistosome-malaria coinfection. In human studies where measures of immune polarisation have been included there have been no concurrent measurement of markers of schistosome stage such as presence and severity of liver fibrosis or urinary tract pathology (Ateba-Ngoa et al., 2015; Courtin et al., 2011; Diallo et al., 2010; Lyke, Dabo, et al., 2012; Lyke et al., 2018; Lyke Kirsten et al., 2006; Lyke, Wang, et al., 2012; Oladele et al., 2014; Reilly et al., 2008; Remoue et al., 2003; Sangweme et al., 2010; Tokplonou et al., 2020; Wilson et al., 2008, 2009). This means conclusions cannot be drawn from these studies about the effects of schistosome stage and the associated immune profile on malaria. One way to bridge this gap in knowledge is with mathematical modelling. Within-host models of blood stage malaria have been used since the publication of the “classical” model in 1989 (Anderson et al., 1989) to investigate various factors affecting disease outcomes. To replicate the synchronised rupture of infected erythrocytes requires the use of multi-compartment models, delay differential equations or partial differential equations (Khoury et al., 2018). Agent based modelling, is not only able to replicate this synchronisation but also allows the duration of infection for each erythrocyte to be tracked. This means that individual erythrocyte-immune cell interactions can be altered over the course of an erythrocyte's infection, by allowing the immune cell to assess how long each erythrocyte has been infected. This mimics the changing visibility of infected erythrocytes to immune cells, resulting from alterations in the erythrocyte cell membranes over the course of a cell's infection (Chan et al., 2014; Gomes et al., 2016).

Using an ABM, we explore the response of malaria to schistosomes at different stages of schistosome infection. Specifically, we hypothesise that i) early schistosome infection, characterised by mixed Th1 and Th2 cytokine activity, will result in more rapid clearance of malaria parasitaemia due to early control of malaria by enhanced cell-mediated killing, ii) chronic schistosome infection, demonstrating Th2 polarisation, will result in longer malaria infection and increased infection intensity due to cross-suppression of early cell-mediated immunity and iii) late chronic stage schistosome infection, as defined by mixed Th2 and Treg polarisation, will have the longest malaria infections and highest parasite infected cell count due to moderation of both early innate immunity and later adaptive immunity by regulatory cytokines.

## 2 Method

An agent-based model was developed in Netlogo 6.2.0, and is described following the ODD (Overview, Design Concepts and Details) protocol for agent-based models (Grimm et al., 2006, 2010, 2020).

### 2.1 Purpose

The purpose of this model is to explore the effects of schistosome infection of varying stages (acute, chronic, late chronic) on blood stage malaria, during which infected erythrocytes encounter the cells of the immune system in the secondary lymphoid tissue, mainly the spleen where circulating blood is filtered and pathogen-specific T and B cells are generated (Engwerda et al., 2005).

### 2.2 State variables

The model environment is set up as a 2D landscape of 300 × 300 patches without edge boundaries, (i.e. following the surface of a torus). This model has two types of entities: erythrocytes and immune cells. Erythrocytes are modelled as patches, with each patch being either a single erythrocyte (which may be infected or uninfected), or an unoccupied space, such that, of the 90, 000 pixels in the model environment, 50, 000 are erythrocytes and the remainder are unoccupied patches. This approximately equates to the erythrocytes found in 0.01 μl of human blood (Dean, 2005). Distribution of erythrocyte patches in the model environment is randomly generated at the start of the model. The age of individual uninfected erythrocytes is not explicitly modelled, but birth and death rates are set such that average lifespan is 115 days (Franco, 2012b). In the model, patches which are designated as erythrocytes therefore a chance of dying equal to 1/(115×24)= 3.62E-4 per hour, with each iteration of the model representing 1 h. Upon death, that patch is converted from an erythrocyte patch to an unoccupied patch. Each hour, 50, 000 patches (a number equal to the total starting erythrocyte count) have a chance of converting into an erythrocyte of 1/(115×24)= 3.62E-4 per hour. Therefore, in the absence of malaria infection, the population of erythrocyte patches will be stable, barring stochastic variation. Infected erythrocyte patches have an additional state variable counting the current stage of intraerythrocytic development (i.e. hours since merozoite invasion).

Immune cells are modelled as individuals (agents) that move over patches in the model environment. In the model, three agent populations are used to represent the diverse cell types and myriad of immunological processes associated with three major functions of immunity, namely the cell-mediated, humoral and regulatory arms of the immune system. These are named Agent 1 (cell-mediated), Agent 2 (humoral) and Agent 3 (regulatory). Global variables in this model are the cytokine concentrations, which are calculated according to equations given in *2.6. Model rationale and submodels.*

### 2.3 Process overview and scheduling

**Fig. 1:**
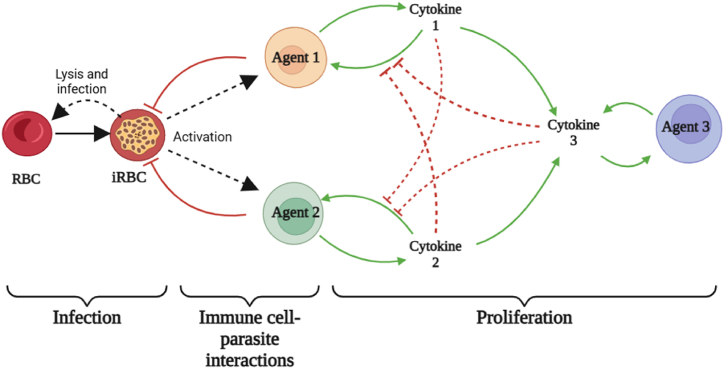
Diagram of interactions in the model. Processes are subdivided into three phases: Infection, Immune cell-parasite interactions and Proliferation. Solid black lines represent transformation between population types. Solid green lines represent upregulation, dashed red lines represent downregulation. Dashed black lines represent an interaction between two cell types, which is noted. Solid red lines represent killing. RBC = red blood cell/erythrocyte, iRBC = parasitised red blood cell/erythrocyte. Created in BioRender.com.

The model iterates in time steps representing 1 h. For each iteration of the model, three phases proceed in order (see Fig. 1), following which the time counter increases by one and the model repeats. Each full model run proceeds for a maximum of 12,000 iterations, or 500 days. The model run terminates earlier than 12,000 iterations if the infected erythrocyte count reaches zero. This time scale has been selected in line with data on both experimentally-induced malaria and modelling of naturally occurring malaria infections. Experimentally-induced malaria was used as a syphilis treatment from the 1920's to 1950's (Collins & Jeffery, 1999) and a subset of individuals who did not receive any antimalarial drugs was found to have a mean infection duration of 130 days (Henry et al., 2022). Patients in that study were atypical, as they were malaria naïve individuals with active syphilis and infected with malaria strains selected for low virulence. It is estimated naturally occurring malaria infections in endemic areas would skew towards a shorter average timescale. This is supported by studies examining natural infections in Malawi (Buchwald et al., 2019), Ghana (Bretscher et al., 2011) and Papua New Guinea (Bruce et al., 2000), where infection duration distribution was positively skewed, with most infections cleared soon after infection (within 50 days) and a minority persisting for a long time, up to 700 days in one model (Bretscher et al., 2011). An initial timescale of 500 days was therefore chosen for our model to balance the likely distribution of malaria infection duration and computational time required for prolonged model runs.

A diagram of interactions between erythrocyte patches and the three immune agent populations in this model are shown in Fig. 1. Birth and death of each cell type are not shown but take place at the start of each model iteration, with birth and death rates set so that the cell populations will remain steady in the absence of malaria infection. Processes are divided into three phases, and run in the following order during each iteration:

1. Infection: If a patch is currently infected, the age of that infection is increased by 1 at the start of each time step. At age 48, an infected patch will die, causing that patch to be converted into an empty space patch and up to 16 uninfected patches in a radius of 2 will become infected. This simulates the 48 h cycle of synchronised lysis seen in *P. falciparum*, and the average infection rate of new erythrocytes by merozoites released from the lysed cells (Tuteja, 2007)
2. Immune cell-parasite interactions: all three immune cell types move across patches. In a single iteration of the model they can move a distance equal to 60x their length. They can move backwards, over a patch they have already travelled over. This models the movement of immune cells through lymphoid tissue, where infected erythrocytes interact with the immune system during blood stage malaria. This is in keeping with a speed of 11 μm/min previously reported (i.e. moving approximately 60x their own length of ∼10 μm over the course of an hour) (Beauchemin et al., 2007). Agents are able in this way to interact with multiple patches during each timestep of the model. If an infected patch (i.e. malaria infected erythrocyte) is encountered the following interaction will take place:
  a. An Agent 1 (A1) cell will have a chance to kill the infected patch. This chance is at its lowest when the patch is newly infected and increases with increasing patch infection age. Between the patch infection ages of 1 and 40 the increase is linear, ranging between 0% and 95%, with a fixed 95% chance where a patch's infection age is above 40. Invaded erythrocytes are essentially invisible to the immune system but as parasite derived antigens are increasingly expressed on the cell membrane surface as infection progresses, the immune system is better able to recognise infected cells, although this doesn't reach a 100% chance (Chan et al., 2014; Gomes et al., 2016).
  b. An Agent 2 (A2) cell will have a chance to kill the infected patch if sufficient time in the model has elapsed for onset of antibody-mediated immunity to have taken place. This time delay has been estimated from as little as 8 to as much as 20 days (Camponovo et al., 2021; Dietz et al., 2006) following detectable parasitaemia and in our model is set at 12 days. The chance of killing the infected patch is calculated the same way as for Agent 1 cells.
3. Proliferation: Each agent type produces a cytokine, which is the means by which agent types upregulate their own proliferation and downregulate others. The cytokine level for Agent 1, Agent 2 and Agent 3 (A3) is calculated during the proliferation step of the model (calculations provided in section 2.6*. Model Rationale and Submodels*). To prevent agent proliferation in the absence of infected cells, the initial population of Agent 1 and 2 do not produce cytokines until after they recognise an infected cell for the first time, at which point they are considered activated and are incorporated into the cytokine calculations. Additional Agent 1, Agent 2 and Agent 3 are generated according to the balance of upregulatory and downregulatory cytokines (see *2.6. Model Rationale and Submodels* for cytokine and agent proliferation calculations). Agents generated by proliferation are considered activated from the time of generation and can contribute to cytokine calculations immediately.

Following each of the three processes described above, the time step counter increases by 1 and the three phases of the model run again.

### 2.4 Design concepts

For model analysis, population level variables were recorded for each patch and agent type, as well as time to resolution of infection (i.e. until infected patch count is zero).

### 2.5 Initialisation and input

During model set up, 20 random erythrocyte patches are designated as infected, equivalent to 2000 parasites/μl, the threshold at which the majority of malaria rapid diagnostic tests perform well to detect malaria infection (World Health Organization, 2018). The baseline starting population of Agent 1 cells is set at 30 and starting Agent 2 population is set at 20. This results in a bias towards Agent 1 as the earliest response type and therefore earliest cytokine profile generated, as seen in natural infection (Stevenson & Riley, 2004). Regulatory Agent 3 starting populations are set at 5, as Tregs make up only a small percentage of total white blood cells (Wan, 2010). This results in a total of 55 immune agents total at the start of the model, equivalent to 5500/μl, which is within the normal reference range for white blood cells (Bain, 2022). This model is run as the ‘malaria alone’ condition. To model acute, chronic and late chronic schistosome infection coinfections, additional cytokines are added to the baseline starting conditions. These either promote Agent 1 (schistosome-associated cytokine 1, or S_1_), Agent 2 (S_2_) or Agent 3 proliferation (S_3_). Cytokines for each schistosome infection stage were added in the quantities shown in Table 1. These numbers were selected from a range tested as they provide feasible malaria infection durations and parasite densities (*se*e Supplementary Material Figs. 5–7*).* These schistosome-associated cytokines influence the model by contributing to the cytokine calculations found in *2.6. Model Rationale and Submodels*.

**Table 1:** Quantity of schistosome-associated S_1_, S_2_ and S_3_ cytokines added to the model for each simulated schistosome stage.

Schistosome stage	Cytokine		
	S_1_	S_2_	S_3_
*Acute*	5	5	0
*Chronic*	0	10	0
*Late Chronic*	0	5	5

### 2.6 Model Rationale and Submodels

As merozoites have a short lifespan, of only minutes, in comparison to the model time step of an hour and are rapidly degraded if they do not enter and infect an erythrocyte (Blake et al., 2020; Boyle et al., 2010; Johnson et al., 1980), the biological process of merozoite release from ruptured erythrocytes is not explicitly modelled. Instead, up to 16 nearby uninfected erythrocytes are immediately infected. Fewer than 16 cells may be infected if the rupturing erythrocyte does not have that many uninfected cells within a radius of 2 patches.

Cytokines also have a short half-life compared to that of immune cells and so cytokine concentration is calculated each as equal to the sum of the current counts of activated cell types that produce them, rather than modelling each cytokine as individual agents. Total cytokines can be calculated as the sum of the current quantity of their corresponding agent, plus any additional schistosome-associated cytokine, as follows:

$$C_{1}=A_{1}+S_{1}$$

$$C_{2}=A_{2}+{\;S}_{2}$$

$$C_{3}=A_{3}+{\;S}_{3}$$

Proliferation of cells is calculated using Hill-type functions for both the suppressive and proliferative cytokines. Agent 1 production is suppressed by Agent 2 and Agent 3 and so it's proliferation can be given as:

A_1_ produced at proliferation step $=\frac{{Vmax}_{1}\times C_{1}^{\;n}}{k_{0.5}^{\;n}+C_{1}^{\;n}}\times \left(1-\frac{{Vmax}_{2}\times C_{2}^{\;n}}{k_{0.5}^{\;n}+C_{2}^{\;n}}+\frac{{Vmax}_{3}\times C_{3}^{\;n}}{k_{0.5}^{\;n}+C_{3}^{\;n}}\right)$

Agent 2 production is suppressed by both Agent 1 and Agent 3 cytokines and therefore is given as:

A_2_ produced at proliferation step $=\frac{{Vmax}_{2}\times C_{2}^{\;n}}{k_{0.5}^{\;n}+C_{2}^{\;n}}\times \left(1-\frac{{Vmax}_{1}\times C_{1}^{\;n}}{k_{0.5}^{\;n}+C_{1}^{\;n}}+\frac{{Vmax}_{3}\times C_{3}^{\;n}}{k_{0.5}^{\;n}+C_{3}^{\;n}}\right)$

Agent 3 production requires the presence of both Agent 3 cytokine and either Agent 1 or 2 cytokine and is calculated as:

A_3_ produced at proliferation step $=\frac{{Vmax}_{3}\times C_{3}^{\;n}}{k_{0.5}^{\;n}+C_{3}^{\;n}}\times \left(\frac{C_{1}+C_{2}}{{Ck}_{0.5}+C_{1}+C_{2}}\right)$

The suppressive power of C_3_ is greater than the power of C_1_ and C_2_, in line with data on the maximum suppression by TGF-β compared to cytokines produced by other lymphocyte types (Cosmi et al., 2004; Fernandez-Botran et al., 1986; Fiorentino et al., 1991; Gajewski & Fitch, 1988). For values of model parameters, see Table 2 below. Where parameters are derived from previously published data, this is noted. For parameters determined within the current study, an explanation for their derivation is given in Supplementary material.

**Table 2:** Parameter definitions and values.

Parameter	Value	Parameter Origin
Maximum rate of cell proliferation (Vmax)	60	Defined in current study (see Supplementary Material Fig. 1)
Half saturation constant for cell proliferation (k_0.5_)	20	Defined in current study (see Supplementary Material Fig. 2)
Hill coefficient (n)	2	Defined in current study (see Supplementary Material Fig. 3)
Maximum suppression by C_3_ (Vmax_3_)	0.8	(Cosmi et al., 2004; Fiorentino et al., 1991)
Maximum suppression by C_1_/C_2_ (Vmax_1_, Vmax_2_)	0.4	(Fernandez-Botran et al., 1986; Gajewski & Fitch, 1988)
Half saturation constant for C_1_/C_2_ in Agent 3 proliferation (Ck_0.5_)	600	Defined in current study (see Supplementary Material Fig. 4)
Length of infection of erythrocyte	48 h	Tuteja (2007)
Number of new patches infected	Up to 16	Tuteja (2007)
Birth rate of erythrocytes	3.62E-4 per iteration, for the starting population of erythrocytes	Franco (2012a)
Death rate of erythrocytes	3.62E-4 per iteration	Franco (2012a)
Birth rate of Agent 1/2/3 cells (without stimulation)	6E-3 per iteration, for the starting population of agent type	Pepper and Jenkins (2011)
Death rate of Agent 1/2/3 cells (without stimulation)	6E-3 per iteration	Pepper and Jenkins (2011)

In this model, several assumptions are made to provide a simplified framework of Agent 1/Agent 2/Agent 3 interactions. As already discussed, each agent population and their associated cytokines represents an array of cell types, processes and cytokines associated with their respective arm of the immune response, rather than a literal representation of a single cell type. The actions of antigen-presenting cells in processing and displaying antigen from infected erythrocytes to naïve T cells is not explicitly modelled. It is also assumed that each immune cell population is only capable of producing one type of cytokine. While it has been demonstrated *in vitro* that early T helper cells can switch phenotype, this ability is lost once the cell is fully differentiated (Murphy et al., 1996; Zhu & Paul, 2010) and so this process is not included in the model.

### 2.7 Statistical analysis

Each group (malaria alone, acute, chronic and late chronic schistosome coinfection) was run 600 times, for a total of 2400 model runs. Analyses were conducted in R v. 4.4.1 (R Core Team, 2021). The effect of simulated acute, chronic and late chronic schistosome coinfections, on malaria infected cell count and duration of infection were assessed using general linear models (GLMs). Model fit was evaluated by assessment of the residual distribution and AIC. The best fit model for peak malaria infected cell count included a natural log transformation for the response variable, a Gamma error family and an identity link function. For the infection duration model, best fit was achieved with a square root transformation for the response and a gaussian error family and identity link function. Provided the best fitting model. For both models, response variable transformation was conducted prior to model fitting. While the chosen family and link functions provided the best model fits, the residual distribution was non-normal, therefore, the Kruskal-Wallis test was also performed on the data to ensure the robustness of the analyses. Where statistical differences in a term were identified, contrasts (O'Callaghan A et al., 2020) were used to assess the significance of between group differences for the GLMs and the pairwise Wilcox test for the Kruskal-Wallis analyses.

## 3 Results

### 3.1 Malaria infected cell counts

**Fig. 2:**
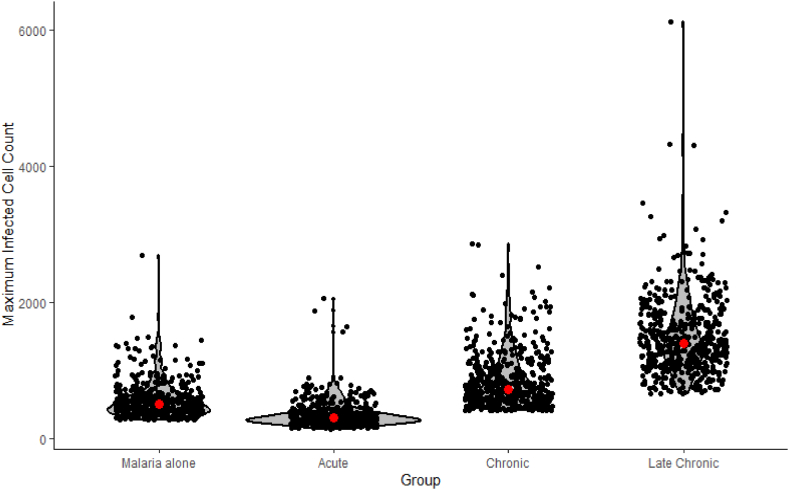
Effects of schistosome coinfection of varying stages on maximum malaria infected cell count. Maximum infected cell count is based on a starting infected cell count of 20 and uninfected cell count of 49,980. The mean cell count is shown in red for each group. Individual points are jittered to aid visualisation and are overlaid on a violin plot of the distribution. N = 600 for each group.

The GLM analysis indicated that the maximum number of malaria infected erythrocytes was significantly affected by schistosome coinfection (F_3, 1996_ = 1357, p < 0.01, model adj R sq 0.67, Fig. 2), but the direction of change was dependent on the stage of the schistosome infection. All stages of schistosome infection were significantly different from one another (Table 3). Peak malaria infected cell counts in acute schistosome infection (358 ± CI 8.6) were 37% lower than in the control (565 ± CI 11.3)*,* while both chronic and late chronic infections resulted in peak infected cell counts that were 52% and 165.7% higher respectively compared to the control (856 ± CI 17.9 and 1501 ± CI 25.2). Kruskal-Wallis with pairwise Wilcox tests agreed with the findings of the generalised linear model (Supplementary Table 1).

**Table 3:** Comparison of GLM outcomes for (log) peak infected cell count between groups.

	Contrast	CI (upper - lower)	T value
**Control – Acute**	0.47	0.51–0.43	19.81
**Control – Chronic**	−0.40	−0.35–−0.45	−16.85
**Control – Late Chronic**	−0.99	−0.94–−1.03	−41.63
**Acute – Chronic**	−0.87	−0.83–−0.92	−36.66
**Acute – Late Chronic**	−1.46	−1.41–−1.51	−61.44
**Chronic – Late Chronic**	−0.59	−0.54–−0.63	−24.78

### 3.2 Duration of infection

**Fig. 3:**
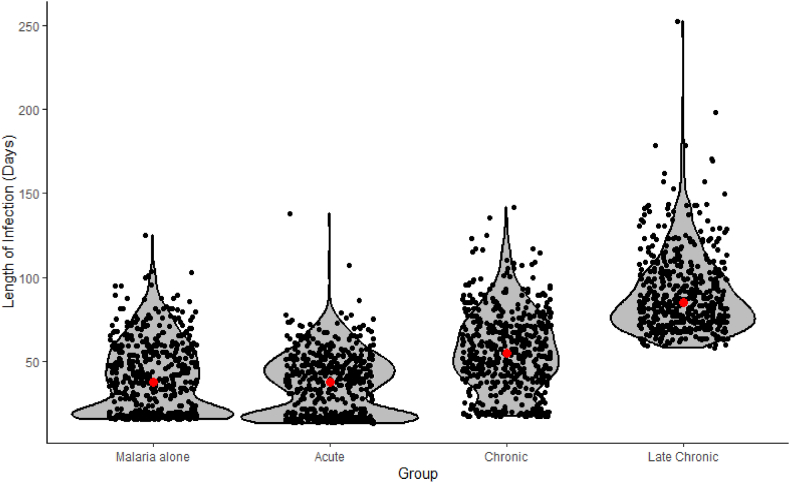
Effects of schistosome coinfection stage on duration of malaria infection. The mean duration is shown in red for each group and points have been jittered to aid visualisation and are overlaid on a violin plot of the distribution. N = 600 for each group.

The duration of malaria infection was also significantly and differentially altered by the stage of the schistosome infection (F_3, 1996_ = 566.4, p < 0.01, model adj R sq 0.65, Fig. 3). For all infection stages, groups differed significantly from one another (Table 4). The acute schistosome infection group had the shortest mean infection duration (36.8 ± CI 0.8 days), followed by the control group (40.6 ± CI 0.9days), then the chronic infection group (55.7 ± CI 1.08 days). The late chronic infection group was markedly longer, with a mean duration of 90.7 ± CI 1.1 days. Kruskal-Wallis with pairwise Wilcox tests agreed with the findings of the generalised linear model (Supplementary Table 2).

**Table 4:** Comparison of GLM outcomes for (sqrt) duration of infection between groups.

	Contrast	CI (upper – lower)	T
**Control – Acute**	0.27	0.46–0.08	2.80
**Control – Chronic**	−1.13	−0.94–−1.32	−11.69
**Control – Late Chronic**	−3.31	−3.12 to −3.50	−34.20
**Acute – Chronic**	−1.40	−1.21–−1.59	−14.48
**Acute – Late Chronic**	−3.58	−3.39–−3.77	−22.51
**Chronic – Late Chronic**	−2.18	−1.99–−2.37	−22.51

**Fig. 4:**
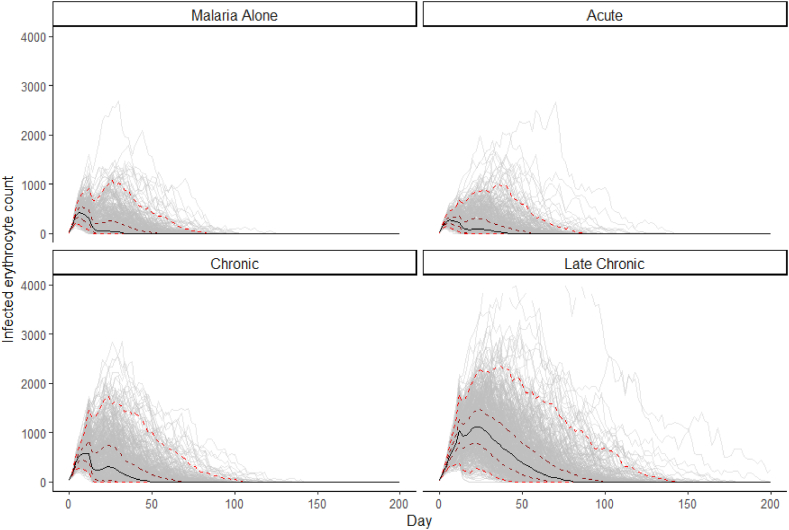
Plot of simulated model data for each group (N = 600), where the black solid line represents the median, and red dotted lines represent the 2.5–97.5 percentile (outer lines) and 25–75 percentile (inner lines). A minority of data points in the late chronic group fall outside the displayed axis limits and have been truncated for visualisation purposes, however all data was used in quartile calculation.

All groups in our model had a range of malaria infection durations. The control group had a range of 15.5–125 days, although this was positively skewed, with only around 30% of infections lasting longer than 50 days. Time plots of the course of infection in each group are shown in Fig. 4.

## 4 Discussion

The output from our model supports the hypothesis that the immune profile associated with early stage schistosome infection is associated with reduced malaria infection intensity and reduced duration of infection, while chronic infection is associated with longer durations and higher peak parasitaemia. It is important to note that this model only provides one possible explanation for the within-host interaction between malaria and schistosomes. Interspecific resource competition has also been proposed as a contributing mechanism of malaria-helminth interaction in coinfection, although at present this has only been demonstrated in hookworm (Budischak et al., 2018; Griffiths et al., 2015) and not in other helminth species. Our model currently does not account for resource competition as an additional point of interaction but could be extended to include the impact of competition for erythrocytes in the future.

As previously mentioned, there is a scarcity of real-world data on the effects of schistosome stage on malaria. In endemic areas, children acquire first schistosome infections while still at preschool age (Poole et al., 2014; Stothard et al., 2013; Stothard, Sousa-Figueiredo, Betson, Adriko, et al., 2011). These schistosome infections are likely to be more often in their early stages, as chronic infection, and its associated immune changes, only develops when schistosome eggs begin to accumulate in tissues. It can be inferred, therefore, that younger children are more likely to have early schistosome infections, while older children and adults are more likely to have chronic schistosomiasis. This is supported by comparisons of which arms of the immune system are enhanced in malaria-schistosome coinfection in these age groups. Where immune response have been compared, coinfected children showed increases in cytokines associated with a type 1 immune response (i.e. IFN-γ), while coinfected adults also showed elevation in cytokines associate with type 2 and regulatory immune responses (Diallo et al., 2004). When considering the impact of age on antibody production, one study which subdivided its cohort of children into 4–8 and 9–14 years old found increased IgG antibodies to malaria in coinfection versus single malaria infection but only in the older age group (Lyke, Wang, et al., 2012), which could support the idea that more established schistosome infection may bolster the antibody response.

Clinical outcomes have also been reported to vary with age. In a study of children aged 1–5, our research group found reduced malaria parasitaemia in schistosome coinfected children (McDowell et al., 2022), while other studies have reported the same relationship in children aged 4–8 (Lyke et al., 2005; Tokplonou et al., 2020), but not in older children in the same cohort (Lyke et al., 2005). In contrast, studies with a wider participant age range have reported conflicting results on the effects of schistosome coinfection on malaria. There have been reports of both increased parasitaemia (Degarege et al., 2012; Friis et al., 2000; Getie et al., 2015; Tuasha et al., 2019) and decreased parasitaemia (Briand et al., 2005; Kinung'hi et al., 2014; Lemaitre et al., 2014; Lyke, Dabo, et al., 2012; Lyke, Wang, et al., 2012), as well as studies which did not find a significant difference (Diallo et al., 2004; Hillier et al., 2008; Kamau et al., 2021; Mazigo et al., 2010; Mulu et al., 2013; Oladele et al., 2014; Raso et al., 2004). These conflicting results may be a result of heterogeneity in participant ages, as well as the increased intra-group variability seen in the chronic and late chronic groups in our model (see Fig. 4). In these human studies, age is likely to be an imperfect proxy for schistosome stage, as although chronic infection is less common in young children, both hepatic (Balen et al., 2006; Bustinduy et al., 2014; Davis et al., 2015; Stothard et al., 2013) and urinary tract (Bocanegra García et al., 2018; Garba et al., 2010; Meurs et al., 2012) sequalae of chronic schistosomiasis have also been found in both preschool and school age children.

Animal coinfection trials do not suffer from the issues of unknown infection stage seen in human studies. When the effect of chronic schistosome coinfection on malaria has been studied in mice and primates, the results agree with our analyses, reporting increased malaria parasitaemia levels with chronic schistosome infection (Bucher et al., 2011; Helmby et al., 1998; Legesse et al., 2004; Sangweme et al., 2009; Semenya et al., 2012; Wang et al., 2013, 2014; Yoshida et al., 2000). To further understand if the changing effects of schistosomes on malaria severity are indeed mediated by schistosome stage, future work should focus on determining the chronicity of schistosome infections in study populations. It is especially important to understand if PSAC are benefitting from mitigated malaria severity due to early schistosome infection, since PSAC are now to be included in routine mass drug administration against schistosomes (World Health Organization, 2022), which may lead to the unintended consequence of increased malaria parasite density. Control programmes should therefore consider malaria-schistosome coinfection and integration of treatment programmes.

Alongside increased malaria parasite density, our model shows longer malaria infections in chronic schistosomiasis, in comparison to both the malaria single infection and schistosome acute infection group. Increased malaria infection duration is demonstrated in chronic schistosome coinfection in animal experiments (Legesse et al., 2004; Sangweme et al., 2009; Semenya et al., 2012). While duration of natural malaria infection is difficult to study directly in human participants, increased prevalence of malaria has been reported in schistosome positive participants (Degarege et al., 2012; Doumbo et al., 2014; Tuasha et al., 2019). While increased duration of infection will lead to individuals testing positive for longer, it is also possible that increased duration of individual malaria infections could contribute to an increased incidence in a community by prolonging the infectious period and therefore increasing transmission.

Our model provides a simplified framework of the immune system response to blood stage malaria which could be adapted to other species of malaria with differing merozoite counts and erythrocyte cycle lengths. It could also be adapted for other coinfecting species by considering the effects of those organisms on the immune system and altering the Agent 1/2/3 composition accordingly. Further expansions to the model could be implemented to address other key questions such as exploring different outcomes for clinical malaria through immunopathology.

The most severe form of immunopathology in *P. falciparum* malaria is cerebral malaria. Th1 cytokines drive immunopathology and it could therefore be theorised that the augmented Th1 cytokine production in early schistosome infection could increase the risk of cerebral malaria, which is most commonly seen in PSAC. These severe sequalae are rarely seen in coinfection studies which generally recruit from outpatient and community settings or eliminate those with severe manifestations of malaria such as cerebral malaria from their study population. One study on children aged 1–5 years did demonstrate a sevenfold greater odds of severe immunopathological sequelae, including cerebral malaria, in those coinfected with *Schistosoma haematobium*, despite a lower overall risk of malaria (Mduluza-Jokonya et al., 2020). This supports a role for Th1 leading to enhanced clearance of the malaria parasite in coinfected young children with acute schistosome infections but with the potential for exacerbated immunopathology attributed to this pro-inflammatory response. In animal studies, chronic schistosomiasis, which is associated with Th2 activation co-infection with malaria is associated with attenuation of Th1 cytokines and chemokines and a reduced incidence of cerebral malaria and (Bucher et al., 2011; Semenya et al., 2012; Waknine-Grinberg et al., 2010; Wang et al., 2013, 2014; Yoshida et al., 2000). The risk of cerebral malaria at different stages of schistosome infection will be a future focus as we expand our model.

Onward transmission of malaria is another important aspect of malaria infection dynamics not yet addressed in our model. Gametocytes are the only stage of the malaria life cycle which is transmissible to the mosquito vector and make up only a small proportion of the circulating parasite population. Gametocytogenesis has been demonstrated to be affected by factors such as antimalarial drug treatment (Buckling et al., 1999; Dyer & Day, 2000; Peatey et al., 2009; Price et al., 1996), parasite density (Dyer & Day, 2000; Sondo et al., 2021), host hormones (Dyer & Day, 2000; Lingnau et al., 1993) and the host immune response (Ono et al., 1986; Smalley & Brown, 1981). While coinfection with schistosomes has been shown in children (Sangweme et al., 2010) and mice (Moriyasu et al., 2018) to increase the proportion of gametocytes, the picture is not completely clear, and immune activation has also been demonstrated to reduce gametocyte maturation (Cao et al., 1998) and infectivity to the mosquito (Moriyasu et al., 2018). Further modelling work could aid in shedding light on the effects of schistosome coinfection on gametogenesis.

Another potential expansion of the model concerns schistosome treatment. In schistosome-endemic regions, mass drug administration of praziquantel is recommended either annually or every six months (World Health Organization, 2022). Praziquantel treatment is active against adult worms, disrupting their tegument and exposing worm antigen to immune recognition (Shaw & Erasmus, 1987). This boost to available antigen targets may augment type 2 associated IgG1, IgE, Il-4 and Il-5 (Mitchell et al., 2014; Wilson et al., 2014) and reduce regulatory cytokine Il-10 (Watanabe et al., 2007). By considering the changing immune profile during and in the months following praziquantel treatment this model could investigate optimum timing of malaria treatment around mass drug administration against schistosomes, while also taking into account factors such as suboptimal cure (Sisay et al., 2025) and rapid reinfection rates (Mbanefo et al., 2014; Zacharia et al., 2020).

Overall our work suggests that the stage of infection is important when considering the effects of schistosome coinfection on both the intensity and duration of malaria. Our model suggests that PSAC, who are more likely to suffer from acute schistosome infection, may experience attenuation of their malaria infections. Extension of our model to consider the effects of enhanced Th1 immune responses on immunopathology will help elucidate whether there are consequences to this protection.

## Data access statement

Our agent-based model and the dataset generated from the model and used in this paper are available in Zenodo data repository at 10.5281/zenodo.15496592.

## CRediT authorship contribution statement

**Sarah Rollason:** Writing – review & editing, Writing – original draft, Visualization, Methodology, Investigation, Formal analysis, Conceptualization. **Eleanor Riley:** Writing – review & editing, Methodology. **Joanne Lello:** Writing – review & editing, Supervision, Methodology, Conceptualization.

## Declaration of competing interest

The authors declare that they have no known competing financial interests or personal relationships that could have appeared to influence the work reported in this paper.
