# Supplementary material for: Stage specific immune responses to schistosomes may explain conflicting results in malaria-schistosome coinfection studies
Source: Infect Dis Model. 2025 May 20;10(4):1003–18. doi: 10.1016/j.idm.2025.05.008 (PMC12159226; doi:10.1016/j.idm.2025.05.008)
Supplement: Multimedia component 1 [file mmc1.docx]

# Supplementary Material

For all figures, a local regression line has been added and points have been jittered to aid visualisation.


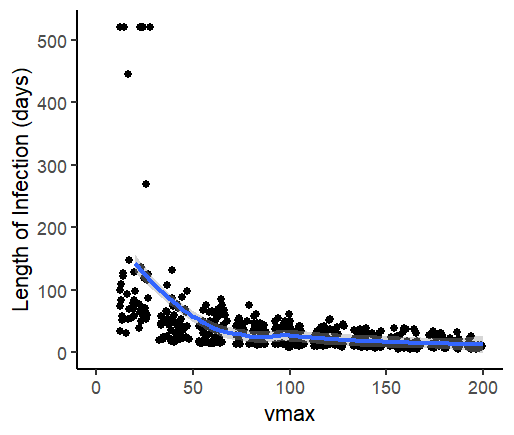


Supplementary Figure 1: Effects of varying the maximum rate of cell proliferation (vmax) within the model. In the final model, vmax is set at 60


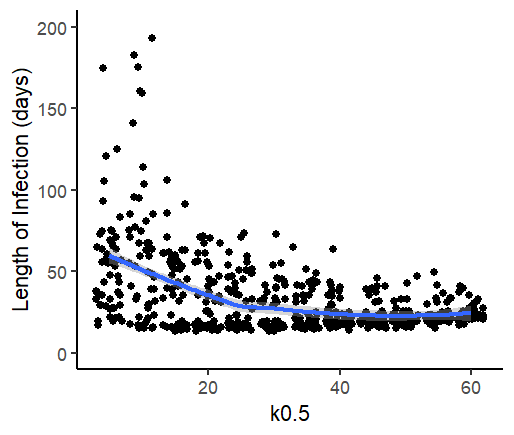


Supplementary Figure 2: Effects of varying the half saturation constant for cell proliferation (k_0.5_) within the model. In the final model, k_0.5_ is set at 20


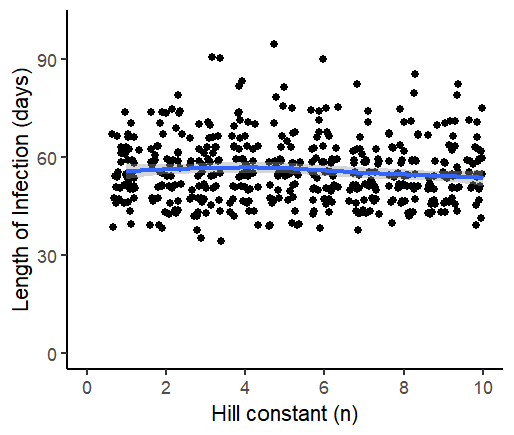


Supplementary Figure 3: Effects of varying the Hill constant (n) within the model. In the final model, n is set at 2

Supplementary Figure 4: Effects of varying the half saturation constant of C1/C2 on Agent 3 (Ck_0.5_)within the model. In the final model, Ck_0.5_ is set at 600


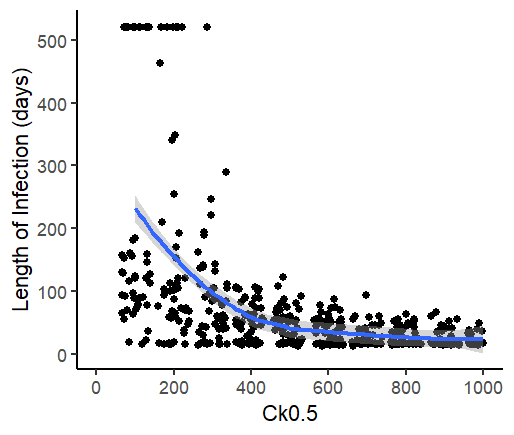

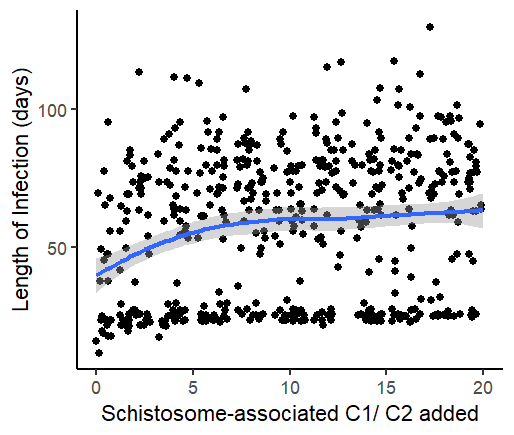

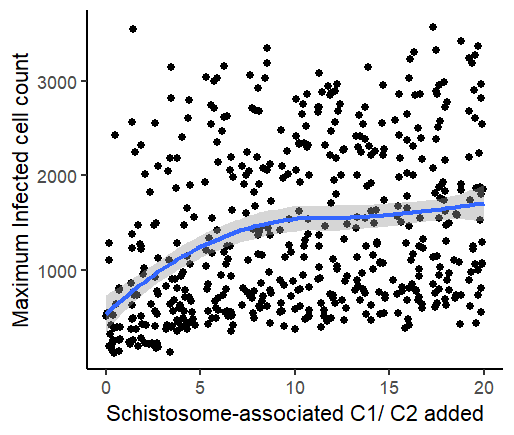


Supplementary Figure 5: Effects of varying the schistosome-associated cytokine 1 and cytokine 2 added to simulate ‘Acute’ schistosome infection in the model. Effects on length of infection (a) and maximum infected cell count (b) are shown. In the final model, the C1 and C2 added for the ‘Acute’ schistosome infection group was set at 5 for each cytokine type, for a total count of cytokine added of 10

(a)

(b)

(a)


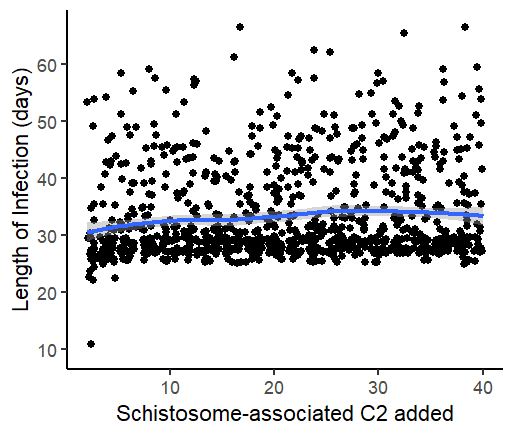

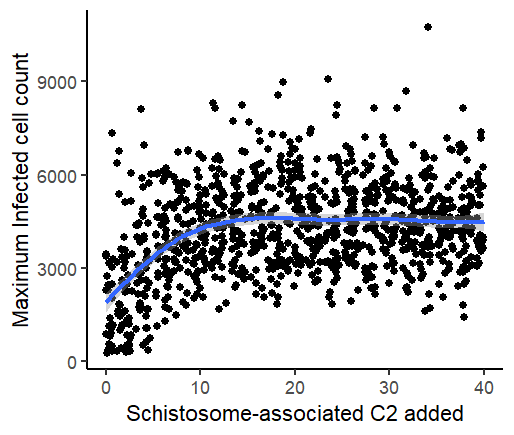


Supplementary Figure 6: Effects of varying the schistosome-associated cytokine 2 added to simulate ‘Chronic’ schistosome infection in the model. Effects on length of infection (a) and maximum infected cell count (b) are shown. In the final model, the C2 added for the ‘Chronic’ schistosome infection group was set at 10

(b)


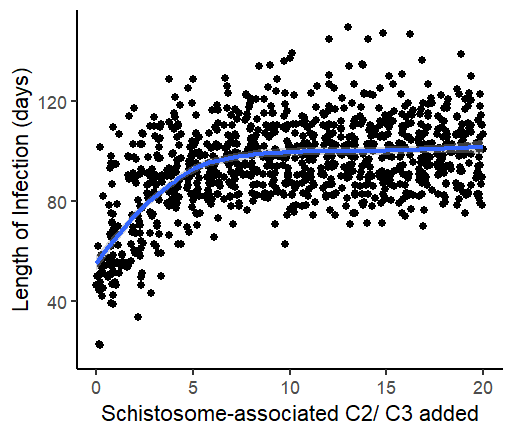

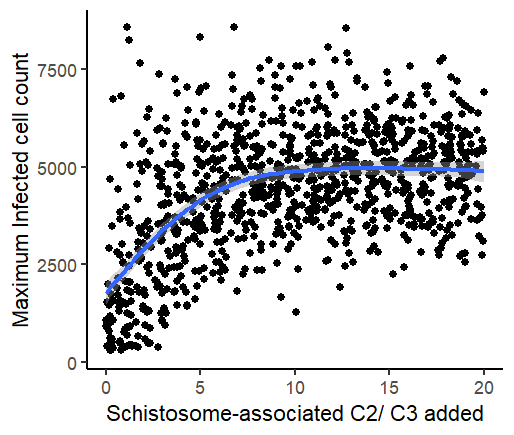


(a)

(b)

Supplementary Figure 7: Effects of varying the schistosome-associated cytokine 2 and cytokine 3 added to simulate ‘Late Chronic’ schistosome infection in the model. Effects on length of infection (a) and maximum infected cell count (b) are shown. In the final model, the C2 and C3 added for the ‘Acute’ schistosome infection group was set at 5 for each cytokine type, for a total count of cytokine added of 10

Kruskal-wallis tests were conducted in addition to GLMs to ensure robustness of analysis when the chosen family and link functions did not result in normal distribution of residuals. Pariwise Wilcox tests were conducted for between group comparisons

Supplementary Table 1: Kruskal-Wallis test with pairwise Wilcox tests was conducted to examine the effects of schistosome coinfection of varying stages on maximum malaria infected cell count. Significant differences were found between groups (Chi square 1366.2, p < 2.2 x10^-16^, df = 3)

|  | **0** | **1** | **2** |
| --- | --- | --- | --- |
| **0** | - | < 2 x10^-16^ | < 2 x10^-16^ |
| **1** | < 2 x10^-16^ | **-** | < 2 x10^-16^ |
| **2** | < 2 x10^-16^ | < 2 x10^-16^ | - |
| **3** | < 2 x10^-16^ | < 2 x10^-16^ | < 2 x10^-16^ |

Supplementary Table 2: Kruskal-Wallis test was conducted to examine the effects of schistosome coinfection of varying stages on duration of malaria infection. Significant differences were found between groups (Chi square 981.86, p < 2.2 x10^-16^, df = 3)

|  | **0** | **1** | **2** |
| --- | --- | --- | --- |
| **0** | - | 0.001 | < 2 x10^-16^ |
| **1** | < 2 x10^-16^ | **-** | < 2 x10^-16^ |
| **2** | < 2 x10^-16^ | < 2 x10^-16^ | - |
| **3** | < 2 x10^-16^ | < 2 x10^-16^ | < 2 x10^-16^ |
